# Barriers to reproductive health care for migrant women in Geneva: a qualitative study

**DOI:** 10.1186/s12978-018-0478-7

**Published:** 2018-03-06

**Authors:** N. C. Schmidt, V. Fargnoli, M. Epiney, O. Irion

**Affiliations:** 1 Department of Obstetrics and Gynecology, University Hospitals of Geneva, Geneva, Switzerland; 2 Department of Sociology, University of Geneva, Geneva, Switzerland

**Keywords:** Reproductive health, Migrant woman, Qualitative study, Focus group

## Abstract

**Background:**

Migrant mothers in developed countries often experience more complicated pregnancy outcomes and less fewer women access preventive gynecology services. To enlighten health care providers to potential barriers, the objective of this paper is to explore barriers to reproductive health services in Geneva described by migrant women from a qualitative perspective.

**Methods:**

In this qualitative study, thirteen focus groups (FG) involving 78 women aged 18 to 66 years were conducted in seven languages. All the FG discussions were audio-recorded and later transcribed. The data was classified, after which the main themes and sub-themes were manually extracted and analyzed.

**Results:**

Barriers were classified either into structural or personal barriers aiming to describe factors influencing the accessibility of reproductive health services vs. those influencing client satisfaction. The five main themes that emerged were financial accessibility, language barriers, real or perceived discrimination, lack of information and embarrassment.

**Conclusion:**

Structural improvements which might meet the needs of the emergent extremely diverse population are the (1) provision of informative material that is easy to understand and available in multiple languages, (2) provision of sensitive cultural training including competence skill for all health professionals, (3) provision of specifically trained nurses or social assistance to guide migrants through the health system and (4) inclusion of monitoring and evaluation programs for the prevention of personal and systemic discrimination.

## Plain English summary

Migrant mothers in developed countries often experience more complicated pregnancy outcomes and fewer migrant women access preventive gynecology services. The objective of this paper was to explore barriers to services for reproductive health (RH) in Geneva described by migrant women from a qualitative perspective. The study used a qualitative approach and thirteen focus groups, involving 78 women aged 18 to 66 years, conducted in seven languages. The data was analyzed, and common themes were identified.

The five main barriers that emerged were financial accessibility, language barriers, real or perceived discrimination, lack of information and embarrassment.

In conclusion, the study suggested the following four interventions to reduce barriers for migrant women to reproductive health care services:

1. The provision of informative material that is easy to understand and available in multiple languages.
2. The mandatory provision of sensitive cultural training for health professionals.
3. The provision of specifically trained nurses or social assistance to guide migrants through the health system.
4. The inclusion of monitoring and evaluating programs for the prevention of personal and systemic discrimination.

## Background

Migration is increasing worldwide. Migrants move to high-income countries for a variety of reasons. On the one hand, individuals migrate to improve their employment opportunities (so-called labor migration), while on the other hand, individuals are forced to migrate due to conflict, human rights violations or persecution. The International Office of Migration reported in 2015 that more than one billion people in the world are migrants, among which 48% are women [1]. In Switzerland, the foreign-born population has greatly increased, and the canton of Geneva is among those with the highest migrant population. In 2016, 41% of the residents in the canton of Geneva had a nationality other than Swiss [2].

While a diverse population bears important chances for economic development, inequalities in pregnancy and childbirth outcomes and disparities in access to gynecology services between migrants and non-migrants have been reported internationally [3–5]. This finding is in accordance with studies conducted in Switzerland, which described a higher maternal and infant mortality among women with a non-Swiss nationality in comparison to their Swiss peers [6–8]. Furthermore, newborns of mothers, especially those from Africa or South East Asia, have been reported to have lower birth weights and to be more frequently transferred to the neonatal unit [6, 9]. In the field of gynecology, studies in Switzerland described more voluntary abortions among women with a non-Swiss nationality, higher numbers of unwanted pregnancies among undocumented migrants compared with women with a legal resident permit or Swiss women, as well as lower screening rates for cervical or breast cancer in some ethnic groups [10–12].

Reasons for health disparities among migrants and the population of the receiving country are multi-factorial and often difficult to disentangle. Published literature has stated, among other factors, challenges in accessing the health care system (such as language barriers, lower health literacy, and low trans-cultural proficiency of health care providers) but also socio-economic difficulties or cultural beliefs [4, 13, 14].

Engaging communities in health care interventions to reduce barriers or stigma can present a unique mode to deliver care with the potential advantage of improving individual’s health. Community health programs, either in ethnic communities or in faith-based organizations, have been implemented with notable success, among others, in the United States of America [15–17].

In Switzerland, little is known about whether communities for migrant women support their members in terms of understanding and accessing the reproductive health care system. Therefore, the aim of our study was to identify barriers to access reproductive health services by migrant women in Geneva and to understand if the community played a role in addressing those barriers.

## Methods

### Study design

A qualitative methodology was used. Qualitative methods are commonly chosen as appropriate to capture the views of marginalized groups such as migrants or the perspectives of females [18, 19]. Between April 2014 and June 2015, thirteen focus groups (FG) were conducted to describe the experiences and perceptions of reproductive health-related events and to find commonality and identify differences with other participants. The semi-structured interview guide started in a supportive and non-judgmental way with general health topics about the comprehension of women’s health, such as women’s behaviors to maintain good health, and evolved to more specific topics such as negative experiences with RH services.. Core topics included i) the comprehension and promotion of women’s health, ii) health-seeking behavior, iii) experience with reproductive health services in Switzerland and home country, iv) information, and v) the role of the community.

### Sampling

The study used the approach of systematic, non-probabilistic sampling. According to the standards of qualitative methodology, we applied the principle of saturation and considering time and access, we aimed to recruit approximately 80 women who identified themselves as a migrant and as a member of a community.

We used multiple recruitment strategies to access the communities, including personal contact of the migrant by email or telephone, making announcements at community gatherings and using the snowball-method [20]. In some situations, a flyer in the maternal language of the targeted participants was used to provide additional information and to determine interest and eligibility. Interested communities participated in an initial meeting that supplied further information about the study. Once a community decided to participate, selected community members reviewed the FG guidelines and assisted with the translation. When interested, community members were trained to moderate the FG in the participant’s language. Community members recruited the participants, and interviews were either organized in the community or in a private room provided by the hospital.

We recruited participants from communities that have been previously described as either disadvantaged or most rapidly growing foreign nationalities in Geneva city. Therefore, we included the perspective of women from Eritrea, Albania, the Philippines, the Middle East and Latin-America. Women were recruited from migrant communities that were active in different areas such as ethnically related groups, religious communities and language schools. To be eligible, participants had to identify themselves as migrants and to be aged 18 years or older. We used the concept of self-defined ethnic identity, which reflects the national identity, but also the social environment in which one interacts such as family, employment or community [21, 22].

### Interview process and data collection

FGs were conducted either in French, English or the maternal language of the participants. An experienced facilitator, one out of two investigators of the research team, moderated all discussions: one medical doctor (NS) and one sociologist (VF). The investigators collaborated during the different phases of the project with the community members; for example in the translation of the study guide and interpretation of results.

Community members served as translators, or if they preferred to participate in the FG, participants identified by the group accepted a translator. All FGs were digitally audio-recorded, transcribed and translated into French (two English FG were transcribed directly into English). Translated transcripts were read through multiple times, summarized and then thematically coded using the qualitative analysis software ATLAS.ti CAQDAS. Most codes were defined in advance according to the main research questions, and additional codes emerged during the coding process itself. A snack was provided at the end of the FG, and participants received a reimbursement of a maximum of twenty Swiss Francs for their participation, depending on the decision made by the community.

### Study setting

The study was conducted in Geneva city, which is the most populous city in the French-speaking part of Switzerland. The canton of Geneva has a total of 493,706 inhabitants (2016). A large number of international organizations have their headquarters and agencies in Geneva, and in 2015 41% if the canton inhabitants were of a non-Swiss nationality. Gynecology or obstetric services are provided at the University Hospital of Geneva, a public hospital, or in private cabinets or clinics in Geneva. This study was initiated at the Department of Obstetrics at the University Hospital of Geneva and was approved by the Ethical Review Board of the Canton of Geneva (CER 14–095).

## Results

Between April 2014 and June 2015, thirteen FGs in six communities were conducted (Table 1). A typical FG lasted between 90 and 150 min. The average age of the participating women was 41 years (range: 18 to 66 years), and the majority (57.7%) of the women were married. Participants were from 23 countries, and on average, the women had lived in Switzerland for nine years (range: 3 months to 25 years). Their education level was good, with 84.6% having at least a college degree. In addition, every sixth participant reported having no health insurance (15.4%). The socio-demographic characteristics are shown in Table 2.

**Table 1:** Details about focus groups

Number of FGs	Number of participants	Continent of origin	Language of FG	Number Community
	*3 FG test (English and French)*			
1	7	Middle East	French	1
2	5	Latin-America	Spanish	2
3	7	Latin-America	Spanish	2
4	7	Middle East	Persian	1
5	6	Africa	Tigrinya	3
6	4	Latin-America	Portuguese	4
7	7	mixed (Europe, Africa, Latin-America)	French	5
8	5	mixed (Europe, Africa, Middle-East)	French	5
9	6	mixed (Europe, Africa, Middle-East)	French	5
10	5	mixed (Europe, Africa, Middle-East, Latin-America)	French	5
11	8	Europe	Albanian	5
12	5	Asia	English	6
13	4	Asia	English	6

**Table 2:** FG participants characteristics

	Focus group participants (*n* = 78)
Women 18–66 years	78 (100)
Mean age in years (SD)	40.96 (12.5)
18–39 years	33 (42.3)
> = 40 years	41 (52.6)
no answer	4 (5.1)
Religion	
christian	30 (38.5)
moslem	33 (42.3)
other	2 (2.5)
no answer	13 (16.7)
Mean years in Switzerland	9.05
< = 3 years	24 (30.8)
4–10 years	30 (38.4)
> 10 years	24 (30.8)
Martial status	
single	15 (19.2)
married or in partnership	45 (57.7)
divorced or separated	11 (14.1)
widowed	5 (6.4)
no answer	2 (2.6)
Children	
yes	57 (73.1)
no	19 (24.4)
no answer	2 (2.5)
Education	
never attended school or <= 6 years	12 (15.4)
finished secondary education	28 (35.9)
some technical school	5 (6.4)
bachelor’s degree or higher	26 (33.3)
no answer	7 (9.0)
Work situation	
part or full-time work	27 (34.6)
not working	38 (48.7)
student	3 (3.8)
incapacity to work	6 (7.7)
no answer	4 (5.2)
Health insurance	
yes	63 (80.8)
no	12 (15.4)
no answer	3 (3.8)

The study had the overarching objective of exploring barriers to reproductive health services and understanding if the community played a supportive role. The results from the data analysis revealed five broad themes that had a major impact on migrant women’s access to reproductive health services. The identified findings were classified according to Higginbottom and colleagues’ recent paper into either factors influencing the accessibility of reproductive health services vs. factors influencing client satisfaction, and categorized as either structural or personal barriers [23].

### Factors influencing the accessibility of reproductive health services

#### Language barrier

In all FGs, the language barrier was one of the main obstacles to accessing care. All but one woman were raised outside of Switzerland, and French was not their primary language. Nearly one-third of the participants expressed their preference for a physician speaking the same language. Esther (Middle East) explained: *My gynecologist…she is Iranian, we speak the same language, so I didn’t have a problem.* Those feelings were shared by other women such as Katia (Latin America): *In my case for example, every time I go to the hospital or to a doctor, I ask for a Spanish speaking doctor. I come around with French. I am not saying that I speak very well. But for me, it is very important to express my feelings in my language because it is not the same to say it in another language that is not understood in the same way….*. Women described their inability to speak French as a major source of anxiety, and they feared misinterpretation or misunderstanding. Amanda (Latin America) explained: *She (the doctor) searched for someone who spoke Portuguese to translate for me. This was what I needed. From this moment on, I felt quieter and I started to understand. T*he provision of a skilled interpreter is a service that is provided at the Geneva University hospital free of charge. However, few participants had relied on those professional interpreter services mainly due to a lack of awareness of its existence or due to the belief that costs would emerge. Mainly family members or friends served as interpreters, and most of the participants experienced the services of a family member as an interpreter as sufficient. How3ever, some women recognized the difficulties of unskilled interpreters; especially in complicated medical situation. Participants reported that family members or friends who served as interpreters were not able to correctly translate either due to difficulties in translating the medical vocabulary or due to their personal emotions. Saba from Eritrea shared her experience while in tears: *The doctor asked if I was in pain. I tried to answer but there was a huge problem of communication [then] I started to cry because I felt frustrated. I asked her to stop touching me and she called my husband. She explained things to him but he didn’t understand because medical explanations are too much for him […]. If an interpreter were there, everything would have been easier».*

In only one of the thirteen FG did the interviewees experience the interpreter as an intruder: *It’s difficult to talk about gynecology issues in our culture […]. It is a taboo, the fact that we are embarrassed. And now, on top of that, there is the interpreter. I mean, it is already difficult to talk about the problem to doctors, but we will probably not see him again. But the interpreter is part of our community. So, there is more chance to cross him again* (Solomon from East-Africa).

#### Lack of information

It is important to receive information to understand the health services, which are provided in the host country. Therefore, the lack of information appeared as the second main barrier expressed by participants. Two different reasons emerged: either because participants did not understand the provided information due to language barriers or because they never received any information. Several participants mentioned that they consulted with a gynecologist only in the case of medical problems, which was sometimes due to time or financial constraints (see financial acceptability), but often women were not aware of available preventive health services. Eva from Peru explained: *Only if we have a problem we start to look for a gynecologist. If we are not sick, we do not think about it*. In nearly all FGs, women shared such experiences: *I only go there [the hospital] to give birth. Otherwise, I do not know any controls (Melete from East Africa).*

Participants who migrated from countries where prevention is less known and provided stated that it would have been helpful for them to receive information about preventive services from their health care providers. Afra (East-Africa) stated: *Until today, my general practitioner didn’t give me any information on preventive check-ups. I think it would be helpful if he could remind us about the controls, especially gynecology check-ups, which are available.*

Women perceived a form of information as crucial to better understand, access and utilize reproductive health care services. Several women agreed with Afra that health care providers should provide them with the essential information, especially about available preventive services such as cervical cancer screening or family planning advice. However, women also recognized the limitations with respect to the time and workload of their doctors: *I prefer it if the gynecologist explains everything to me, but sometimes he might be tired by his work and forget to do it (Woman from Kosovo).*

In addition to the information provided orally by direct contact with health or social professionals, the provision of written information material in multiple languages was rated in almost all FGs as essential to facilitate access and navigate through the Swiss health system. Participants identified different means such as the radio, the television or written information received by mail. However, the two most frequently used sources were either the Internet or health-related sessions in communities or language classes. Importantly, nearly all participants had access to a computer and identified the Internet as a valuable source of information, especially the opportunity to search for information in their maternal language.

#### Financial acceptability

Nearly half of the participants identified costs as the third most important barrier to health care. Even if participants had health insurance, which is mandatory in Switzerland, the costs of high deductibles or co-payments were often mentioned as a barrier to visiting the doctor, which was especially the case for preventive services. Angela (South-Asia) stated: *… going to the hospital is quite expensive. And then so, …. For two years, I have not visited a doctor.*

#### Embarrassment

Embarrassment emerged as a personal barrier on the participant (user) side explaining why women did not access especially preventive gynecology services such as cervical cancer or breast cancer screening. Most women perceived pelvic and vaginal examinations for cervical cancer screening as inconvenient and often painful. Juana from Latin America explained: *When they took the smear it was terrible for me. All afternoon, I had horrible pain and was bleeding*. Physical discomfort is not a barrier related specifically to immigrant women and has been mentioned by Swiss women as well [24]. However, in more than half of the FG discussions, the participants explained that they grew up experiencing the female body as taboo. Veronica from Latin America stated: *We are not taught to discover our bodies…*. Elsa, a mother of two children from Africa explained: *Especially with respect to gynecological problems but also with respect to other health problems, we try to keep it [the problem] to ourselves as long as possible, and if it is a gynecological problem, we prefer to say that we have pain somewhere else because we feel very embarrassed.*

Furthermore, women experienced discomfort when health care providers were not aware of their traditions. Mahan (Middle East) expressed: *Sometimes it would be helpful if the doctor understands our traditions a little bit. For example, as a woman, we do not like to shake hands with a male doctor. But some doctors do not know this!* Similar feelings were experienced when women could not consult with a female doctor. Senobar explained (Middle-East): *They suggested to me to see a male doctor. I didn’t go!* I*n* more than half of the FGs, participants specifically mentioned a preference for a female health care provider for gynecology or obstetric consultations.

### Factors influencing client satisfaction

#### Real or perceived discrimination

Nearly one-third of the participants expressed that they felt that they did not receive the same attendance as Swiss women. Importantly, in nearly one-third of the FGs, women expressed feelings of discrimination in their daily lives. Amanda from Latin America explained: *I would love if integration would be easier. I know that it is difficult to migrate here [to Switzerland]… But it should be easier,…., that people would have less fear. Because of the fear of losing your residence permit, everything is very complicated;* Real or perceived discrimination in the health sector was rarely mentioned with respect to the doctor-patient relationship; it was mainly expressed concerning reception at the registration desk prior to the clinical appointment: *As soon as we arrive, we feel degraded. They want our papers, for example, proof of a health insurance. Even if we have all the papers, they put us in an uncomfortable position. We do not feel welcome. (*Armani from East-Africa).

A few women expressed that waiting time as well as perceived impolite treatment by health professionals were due to their origins or language barriers. Susanna, a women from Latin-America, gave the following example: *And when I was hospitalized, I saw that the doctor spoke a lot more with women who spoke French or were Swiss. And with me, tac, tac, tac. He didn’t have the time to explain to me all that I needed. This disturbed me.*

#### Perceived inadequate provision of health services

In addition to the perceived lack of adequate culturally competent care (see embarrassment), women experienced the process of adhering to appointments for regular obstetric or gynecology visits as very difficult. In several cases, they had to wait several weeks for their appointments, and they complained of long waiting times in the case of emergency situations. Tiba (Middle-East) explained: *I had already had to go several times to the emergency department, but even if I am dying, I will not go there again because I had to wait six hours with a fever, but no one was interested.*

### Strategies to overcome barriers at the community level:

In none of the participating communities were active projects around health established. In four of six communities, health topics, some related to RH (such as gender-based violence and access to cervical or breast cancer screening), had been discussed at least once in the past five years. However, community-based health programs that focused on a specific prevention activity such as cervical cancer screening or healthy behavior promotion over a period of several months were not available. However, importantly, health sessions organized by the community or language schools were especially appreciated by women who had recently arrived or were more illiterate. Afra from East-Africa stated: *It would be nice to have similar meetings such as those today in small groups. This would help us more than receiving letters or flyers.* Furthermore, a community group leader from the Philippines highlighted the importance of educating women from her country to foresee health problems. She recommended the conclusion of a health insurance even in the case of illegal situations to be prepared for emergency health problems as well as to access preventive services*.* Angelica explained: It *is our mentality that we do not want to save something for “rainy days”… It is my dream to educate women from my home country, about what will be necessary when we are getting older. It is like an old car, which might break one day. One part of this education would be that we need insurance*.

In conclusion, in the participating communities, few strategies were utilized to overcome barriers to reproductive health services.

## Discussion

The current study is, to our knowledge, the first with the aim of understanding barriers to reproductive health services among migrants from a qualitative perspective. Five main themes were identified and categorized either in terms of structural or personal barriers: financial accessibility, language barriers, real or perceived discrimination, lack of information and embarrassment (Table 3). In general, our results are fairly consistent with previous national and international literature [3, 15].

**Table 3:** Main barriers identified and related structural improvements

Financial accessibility
• provision of information about structures assisting patients with limited financial resources
Language barriers
• provision and information about interpreter services
Real or perceived discrimination and embarrassment
• provision of cultural competence training for professionals working in the health sector including administrative staff (might reduce embarrassment among women as they do not need to have to explain themselves or their practices)
Lack of information
• provision of information material (including in patient’s maternal language)

However, barriers to health services might vary depending on the kind of services from country to country, but also among different migrant groups. Therefore, it is essential to study structural and personal barriers that are related to organizational behaviors.

In the following discussion, we will specifically address the nuanced findings of the structural barriers that influenced accessibility to or satisfaction with reproductive health services for migrant women because we assume that it might be relatively feasible to target those by health care organizations. Therefore, we will not address barriers such as physical discomfort, which have also been previously for women of the host country or structural barriers at the organizational level such as long waiting times for appointments and during visits. Because even these factors may act as a barrier to the use of such services, they can hinder migrant and Swiss patients equally [3, 24].

First, our study confirmed the recent findings by Higginbottom and colleagues who revealed that although maternity services were equally availably for all members of society, in practice the services were often not accessible to migrant women due to a lack of awareness about their existence [23]. Even if the lack of information could be interpreted as a personal barrier, it should rather be considered, in our opinion, as a structural barrier. Indeed, a health organization that is aware of this situation can provide information to their clients. The lack of information is therefore directly linked to health literacy, which has been defined by the World Health Organization as « the cognitive and social skills which determine the motivation and ability of individuals to gain access to, understand and use information in ways which promote and maintain good health » [25]. As reported previously in two separate studies in Canada and the United Kingdom, the institutional culture of maternity services has been mainly designed for those who understand and can negotiate the system. These studies demonstrated that people who recently arrived and were not familiar with the system faced a range of barriers [26, 27]. These findings are comparable to our study in which migrant women who were either undocumented or in the process of applying for asylum appreciated the system because they were guided by specifically trained nurses or social assistants. Migrants who did not benefit from those services often felt a lack of information to navigate the system and experienced the health professionals as less helpful. Legal migrant women even stated that they would also profit from those services. These positive attitudes towards social workers or migrant health nurses support the merit of including them in the system.

Furthermore, professionals who do not work specifically with asylum seekers were not provided with accurate information concerning how to help migrants and received little assistance to develop a better understanding of the constraints and barriers experienced by this specific social group.

It is also important that information material requires sufficient language proficiency among recipients. In our study, inadequate language proficiency emerged as one of the main barriers to either accession or the on-going use of services. This finding is supported by previous studies that have reported, among others, the preference of immigrants to have same-language physicians [4, 26, 27].

However, even if at the University Hospital of Geneva more than 100 different nationalities are working in the medical and nursing sector, it is an impossible task to provide a same-language physician for every participant. In all FGs, participants indicated that language barriers increased their insecurity about seeking care. Most participants sought out ad hoc interpreters to navigate the system, and our findings revealed that few participants were aware of the provision of free-of-charge skilled interpreters for clients. The few participants who used interpreter services rated the experience positively to influence its future use. Efforts should be undertaken to raise awareness of those services and to provide information about their gratuity as well as the possibility of avoiding certain translators either due to sex or cultural background.

The second structural barrier encountered in nearly all FG and which has been previously described in the literature was the financial accessibility of the system [26]. However, in contrast to other studies, the lack of insurance did not lead to a reported delay in seeking care [4]. This might be because, in 1996, the University Hospital created a health care unit that offers free or low cost medical care to undocumented migrants in Geneva. Even if uninsured migrants are referred to the general services of the University Hospital for gynecological or obstetric care, the health care unit has generated trust among the uninsured migrants over the two decades and reaches the majority of pregnant, undocumented and uninsured women [12].

One important personal barrier influencing access to health services that has been outlined by previous studies is social isolation [4, 23]. Interestingly, this barrier was not encountered frequently in our study, which might be due to two reasons. First, FGs were mainly conducted in ethnic, religious or social groups in which the women had started to create a social network. Second, most of the participants had followed either family members or friends who had already lived for several years in Switzerland. Therefore, those residing longer in the country facilitated the arrival of the new immigrants. This phenomenon also explains why the sub-theme of isolation was mentioned mainly in women who migrated as refugees due to unstable conditions in their home country and arrived in an unfamiliar country.

The last two structural barriers influenced mainly client satisfaction. Challenges in delivery services such as the provision of appointments, waiting times or turnover of health care providers can hinder women’s access to services due to less satisfaction with the services and have been addressed previously [4, 23]. These barriers could be addressed at the organizational level.

One of the most important barriers influencing women’s satisfaction was “real or perceived discrimination”. Crush and colleagues described xenophobia, racism and discrimination as increasingly prevalent in countries that receive a large number of migrants [28]. As Geneva, is one of the cantons with the highest percentage of migrants in Switzerland, this risk might also be present. Xenophobia towards migrants might manifest itself as hostility towards migrants by authorities, neighbors, employers or service providers belonging to the host population [28]. Sometimes perceived discrimination is due to a feeling of inferiority emerging from the restricted rights and entitlements of migrants, which might be especially the case for vulnerable migrants. Furthermore, it has been reported that especially migrants who have migrated due to traumatic circumstances may be more inclined to perceive xenophobia against them [29]. However, this observation could not be confirmed in our study population because migrants with a non-refugee background also frequently expressed real or perceived discrimination. It is important for health care providers and administrative staff to be aware of real or perceived discrimination because it can influence the necessary trusts within the patient-provider relationship and can have negative effects on future health seeking behaviors [29, 30]. In the present study, it was not possible to verify if the staff intended to discriminate. However, efforts should be undertaken to include cultural competence training not only in medical education, as it is already part of the curriculum in Geneva, but also in professional practices for health care professionals as well as administrative staff. Intercultural competence training is a method that aims to enable professionals to communicate effectively and empathically while concomitantly reflecting their own culture and implicit assumptions [31]. Furthermore, it might effectively support patient-professional communications irrespective of the migration background. The inclusion of administrative staff in such training is important to lower the anxiety of participants because they are the first contact with the health system for participants. Even if some authors mentioned that training designed to supply specific cultural knowledge about ethnic groups might risk stereotyping and obscure attention to the needs of women, other studies have also reported that such training improves the abilities of professionals in the health care sector to meet the needs of their patients [31]. In a setting such as Geneva, patient-sensitive competence training might improve patient-provider communication. Furthermore, it might strengthen understanding among health care providers for requests (such as same-sex physicians) that might cause embarrassment among women as they have to explain themselves or their practices, or have to ask for what may be regarded as special treatment.

### Study limitations

While this qualitative study was among the first to explore barriers to RH services in the canton of Geneva and the role of the community, it has several limitations.

First, the qualitative approach of the FG covers the range of issues considered important by the participants. It does not describe the relative importance of the issues. Additionally, the format of the FG might have been influenced by leaders within the groups, despite the efforts of the moderators. Even if we reached thematic saturation in the thirteen-conducted FG in seven different languages, the study utilized convenience sampling for the recruitment of participants through local migrant communities in Geneva city and did not reach those living in more isolated regions who less frequently accessed the city center. Therefore, the results cannot be generalized to all migrant women, and thus future research is required.

Second, the authors of this article do not belong to an ethnic minority group. Even if we tried to reduce those barriers by discussing them and ways to influence them with stakeholders and representatives from migrant communities, the results risk interpretation from a western perspective, which leads to certain ideas about the provision of health care.

Third, the average length of time in Switzerland of our sample was nine years, and therefore this study might be susceptible to recall and temporal effect biases.

Finally, in contrast to other studies, we did not include the provider perspective [22].

## Conclusions

Research examining the reduction of barriers to healthcare services is not only beneficial at the personal and institutional level, but it can also inform health policies and strategies. Especially in times during which a constant influx of new immigrants into Geneva changes the population, efforts of health care providers and public health actors must be continuously renewed and oriented to reach new arrivals. This study identified several structural and personal barriers to reproductive health services in Geneva. Even if the data from qualitative research cannot be generalized to the broader context of Switzerland, many of the findings could potentially be applicable to other countries.

Structural improvements, which might meet the needs of the heterogeneous emergent population, are as follows: (1) the provision of information material that is easy to understand and available in multiple languages, possibly using new mHealth technologies; (2) the mandatory provision of sensitive cultural training for health professionals to inform professionals about the availability of interpreter services and social services to offer appropriate care; (3) the evaluation and adaptation of the skills of nurses or social assistance to guide migrants who are not asylum seekers or who are undocumented through the Swiss health system; and, finally, (4) the inclusion of monitoring and evaluation programs for the prevention of personal and systemic discrimination.

Our study showed that migrant communities are interested in topics about health but provide few activities to support their members. Efforts should be undertaken to involve migrant communities, for example, in the distribution of information and knowledge among newcomers to ensure that they know where to find and how to use the services to which they are entitled.

Such combined efforts could, in our opinion, reduce some of the perceived or experienced barriers on the side of the user.

However, future research exploring community-based health interventions may also be beneficial.

## References

[1] International Organization for Mgration. Global Migration Trends Factsheet 2015. https://publications.iom.int/system/files/global_migration_trends_2015_factsheet.pdf. Accessed 4 Feb 2017.

[2] Bilan et état de la population du canton de Genève en 2016. informations statistiques n° 6 – mars 2017. http://www.ge.ch/statistique/tel/publications/2017/informations_statistiques/autres_themes/is_population_06_2017.pdf. Accessed 25 Jul 2017.

[3] Gagnon AJ, Zimbeck M, Zeitlin J, Alexander S, Blondel B, Buitendijk S, Desmeules M, Di Lallo D, Gagnn A, Gissler M (2009). Migration to western industrialised countries and perinatal health: a systematic review. Soc Sci Med.

[4] Scheppers E, Scheppers E, van Dongen E, Dekker J, Geertzen J, Dekker J (2006). Potential barriers to the use of health services among ethnic minorities: a review. Fam Pract.

[5] Reeske A, Rechel B, Rechel B, Mladovsky P, Deville W (2011). Maternal and child health – from conception to first birthday. Migration and health in the European Union.

[6] Bollini P, Wanner P (2006). Santé reproductive des collectivités migrantes.

[7] Bollini P, Wanner P, Pampallona S (2011). Trends in maternal mortality in Switzerland among Swiss and foreign nationals, 1969-2006. Int J Public Health.

[8] Fässler M, Zimmermann R, Quack Lötscher KC (2010). Maternal mortality in Switzerland 1995-2004. Swiss Med Wkly.

[9] Merten S, Wyss C, Ackermann-Liebrich U (2007). Caesarean sections and breastfeeding initiation among migrants in Switzerland. Int J Public Health.

[10] FOPH What about the health of migrant population groups? What about the health of migrant population groups? 2007 FOPH: BAG GP 8.07 445 d 190 f 155 e 30EXT07011 178893 ISBN 3–905235-65-X.

[11] Moreau-Gruet F, Luyet S, Obsan Bulletin 01/2012 (2011). Population migrante et santé. Analyse des hospitalisations. Analyse de la statistique médicale des hôpitaux et recherche de littérature.

[12] Wolff H, Epiney M, Lourenco AP, Costanza MC, Delieutraz-Marchand J, Andreoli N, Dubuisson JB, Gaspoz JM, Irion O (2008). Undocumented migrants lack access to pregnancy care and prevention. BMC Public Health.

[13] Heaman M, Bayrampour H, Kingston D, Blondel B, Gissler M, Roth C, Alexander S, Gagnon A (2013). Migrant women's utilization of prenatal care: a systematic review. Matern Child Health J.

[14] Bischoff A (2006). Caring for migrant and minority patients in European hospitals. A review of effective interventions.

[15] Minkler M, Wallerstein N, Hall B. Community-Based Participatory Research for Health. 1st ed: Jossey-Bass; 2009.

[16] Gutierrez J, Devia C, Weiss L, Chantarat T, Ruddock C, Linnell J, Golub M, Godfrey L, Rosen R, Calman N (2014). Health, community and spirituality: evaluation of a multicultural faith-based diabetes prevention program. Diabetes Educ.

[17] Fang CY, Ma GX, Handorf EA, Feng Z, Rhee J, Miller SM, Kim C, Koh HS (2017). Addressing multilevel barriers to cervical cancer screening in Korean American women: a randomized trial of a community-based intervention. Cancer.

[18] Barbour R (2008). Introducing qualitative research. A student guide to the craft of doing qualitative research.

[19] Morgan DL (1996). FGs. Ann Rev Sociol.

[20] Ritchie J, Lewis J, Elam G, Ritchie J, Lewis J (2003). Designing and selecting samples. Qualitative Research Practice: A Guide for Social Science Students and Researchers.

[21] Israel BA, Checkoway B, Schluz AJ, Zimmerman MA (1994). Health education and community empowerment: conceptualizing and measuring perceptions of individual, organizational, and community control. Health Educ Q.

[22] Tajfel H (1974). Social identity and intergroup behavior. Soc Sci Inf.

[23] Higginbottom GM, Safipour J, Yohani S, O'Brien B, Mumtaz Z, Paton P, Chiu Y, Barolia R (2016). An ethnographic investigation of the maternity healthcare experience of immigrants in rural and urban Alberta, Canada. BMC Pregnancy Childbirth.

[24] Fargnoli V, Petignat P, Burton-Jeangros C. To what extent will women accept HPV self-sampling for cervical cancer screening? A qualitative study conducted in switzerland. Int J Women’s Health. 2015;(7):883–8. 10.2147/IJWH.S90772 PMC4639555 26604830

[25] WHO. Health Promotion. Track 2: Health literacy and health behaviour. http://www.who.int/healthpromotion/conferences/7gchp/track2/en/. Accessed 25 Jul 2017.

[26] Philimore J (2016). Migrant maternity in an era of superdiversity: New migrants' access to, and experience of, antenatal care in the West Midlands, UK. Soc Sci Med.

[27] Reitmanova S, Gustafson DL (2008). "They can't understand it": maternity health and care needs of immigrant Muslim women in St. John's, Newfoundland. Matern child health.

[28] Crush J, Ramachandran (2010). Xenophobia, International Migration and Development. J Human Dev Capabilities.

[29] Phinney JS, Horenczyk G, Liebkind K, Vedder P (2001). Ethnic identity, immigration, and well-being: an interactional perspective. J Soc Issues.

[30] Ellis BH, MacDonald HZ, Lincoln AK, Cabral HJ (2008). Mental health of Somali adolescent refugees: the role of trauma, stress, and perceived discrimination. J Consult Clin Psychol.

[31] Horvat L, Horey D, Romios P, Kis-Rigo J. Cultural competence education for health professionals. Cochrane Database Syst Rev. 2014;(5):CD009405. 10.1002/14651858.CD009405.pub2. 10.1002/14651858.CD009405.pub2 PMC10680054 24793445

